# Supplementary material for: Rapidly identifying new coronavirus mutations of potential concern in the Omicron variant using an unsupervised learning strategy
Source: Sci Rep. 2022 Nov 9;12:19089. doi: 10.1038/s41598-022-23342-2 (PMC9645309; doi:10.1038/s41598-022-23342-2)
Supplement: Supplementary file 3 — Supplementary Information 3. [file 41598_2022_23342_MOESM3_ESM.pdf]

Supplementary Tables S1-S7

Table S1. Descriptive table of SNV numbers by continent/country/region/sub-region around the world: number of reported viruses, minimum number, 1st quartile, median, mean, 3rd quartile and maximum number of mutations on each viral genome

Table S2. Cross-tabulation of six Omicron clusters with six continents in the world

Table S3. Spactial distributions of four emerging polymutants (R346, A701, I1081, N1192) in the spike protein across all countries around world

Table S4. Spactial distributions of six emerging polymutants (V1887, V2867, Y4852, L5086) in ORF1ab, L106 in ORF3a and D343 in N across all countries around world

Table S5. Haplotype frequencies of joined core haplotypes of polymutants in the spike and non-spike genes.

Table S6. Space-time investigation of newly emerging mutation haplotype "RAINVVFGF" for the first reported case. Note that Canada / Ontario reported three cases in November and one case in December, 2021 without providing actual dates.

Table S7. Space-time investigation of newly emerging mutation haplotype "RAVNVAFGY", that has five mutations andobserved among 60 viruses in United Kingdom.

Table S1

	Continent/Country/Region/Sub-region	Freq	Min	1st	Med	3rd	Max
1	Africa/Botswana/Gaborone	17	14	26	26	30	30
2	Africa/Botswana/Lobatse	1	31	31	31	31	31
3	Africa/Botswana/Otse	1	26	26	26	26	26
4	Africa/Botswana/Palapye	3	30	30	30	30	30
5	Africa/Botswana/South East/Greater Gaborone/Gaborone	52	12	28	30	31	38
6	Africa/Botswana/South East/Greater Gaborone/Lobatse	2	28	28	29	29	29
7	Africa/Botswana/South East/Greater Gaborone/Mochudi	1	29	29	29	29	29
8	Africa/Botswana/South East/Greater Gaborone/Oodi	1	21	21	21	21	21
9	Africa/Botswana/South East/Greater Gaborone/Ramotswa	1	16	16	16	16	16
10	Africa/Ghana/Accra	33	29	29	30	31	32
11	Africa/Malawi/Blantyre	3	30	31	31	32	32
12	Africa/Nigeria/Abuja	11	28	30	30	31	31
13	Africa/Reunion	2	29	29	30	30	30
14	Africa/Senegal/Dakar/Iressef Diamniadio	1	15	15	15	15	15
15	Africa/Senegal/Dakar/IRESSEF DIAMNIADIO	6	29	29	31	33	34
16	Africa/Sierra Leone	1	29	29	29	29	29
17	Africa/South Africa	6	24	26	30	31	31
18	Africa/South Africa/Eastern Cape	23	28	30	30	31	35
19	Africa/South Africa/Eastern Cape/Nelson Mandela Bay	8	28	29	30	30	33

20	Africa/South Africa/Eastern Cape/Sarah Baartman	6	30	30	31	31	31
21	Africa/South Africa/Free State	11	25	29	29	30	32
22	Africa/South Africa/Gauteng	257	18	29	29	30	34
23	Africa/South Africa/Gauteng/City of Johannesburg Metro	2	29	29	30	30	30
24	Africa/South Africa/Gauteng/City of Johannesburg Metro Region A	2	29	29	29	29	29
25	Africa/South Africa/Gauteng/City of Johannesburg Metro Region B	4	29	29	29	29	30
26	Africa/South Africa/Gauteng/City of Johannesburg Metro Region C	6	29	29	30	30	30
27	Africa/South Africa/Gauteng/City of Johannesburg Metro Region E	14	29	29	30	30	32
28	Africa/South Africa/Gauteng/City of Johannesburg Metro Region F	21	29	29	29	30	31
29	Africa/South Africa/Gauteng/Sedibeng	4	28	30	31	31	31
30	Africa/South Africa/Gauteng/Tshwane	27	27	29	30	30	39
31	Africa/South Africa/Kwazulu-Natal	1	23	23	23	23	23
32	Africa/South Africa/KwaZulu-Natal	90	23	29	30	31	34
33	Africa/South Africa/KwaZulu-Natal/eThekwini	42	29	30	31	32	33
34	Africa/South Africa/KwaZulu-Natal/Ilembe	6	29	29	30	31	31
35	Africa/South Africa/KwaZulu-Natal/King Cetshwayo	40	22	29	30	32	35
36	Africa/South Africa/KwaZulu-Natal/Ugu	10	30	30	31	31	33
37	Africa/South Africa/KwaZulu-Natal/uMkhanyakude	3	29	30	30	30	30
38	Africa/South Africa/KwaZulu-Natal/Zululand	13	28	31	31	32	34

39	Africa/South Africa/Limpopo	6	29	29	30	31	31
40	Africa/South Africa/Mpumalanga	35	22	28	28	31	32
41	Africa/South Africa/North West	30	26	29	30	31	32
42	Africa/South Africa/Northern Cape	37	20	28	29	29	32
43	Africa/South Africa/Western Cape	39	27	29	30	30	32
44	Africa/South Africa/Western Cape Province/Cape Town Metro	157	23	29	29	30	33
45	Asia/Bangladesh/Dhaka/Dhaka	2	29	29	30	30	30
46	Asia/Hong Kong	18	29	29	30	31	31
47	Asia/India/Delhi	2	29	30	30	31	31
48	Asia/India/Gujarat/Jamnagar	1	46	46	46	46	46
49	Asia/India/Karnataka	2	30	30	30	30	30
50	Asia/India/Maharashtra/Pune	1	32	32	32	32	32
51	Asia/India/New Delhi	1	30	30	30	30	30
52	Asia/Israel	67	19	29	30	31	32
53	Asia/Japan	7	30	30	32	32	33
54	Asia/Jordan/Amman	2	29	29	30	30	30
55	Asia/Malaysia/Selangor	1	20	20	20	20	20
56	Asia/Maldives/Sandies Bathala resort	1	22	22	22	22	22
57	Asia/Nepal/Bagmati	2	29	29	29	29	29
58	Asia/Pakistan	1	29	29	29	29	29
59	Asia/Singapore	13	29	30	31	31	35
60	Asia/South Korea	9	29	30	30	30	30
61	Asia/Sri Lanka/Marawila	2	21	24	27	29	32
62	Asia/Thailand/Bangkok/Bangrak	1	26	26	26	26	26
63	Asia/Thailand/Bangkok/Nongkhaem	2	32	32	33	33	33
64	Europe/Austria/Burgenland	2	26	27	28	29	30
65	Europe/Austria/Lower Austria	5	29	29	32	33	33
66	Europe/Austria/Lower Austria/Bruck an der Leitha/Schwechat	2	30	30	30	30	30
67	Europe/Austria/Tyrol/Schwaz	1	29	29	29	29	29

68	Europe/Austria/Tyrol/Schwaz/Vomp	3	29	29	29	29	29
69	Europe/Austria/Upper Austria/VÃ¶cklabruck/St. Georgen im Attergau	1	31	31	31	31	31
70	Europe/Austria/Vienna	3	30	30	30	31	31
71	Europe/Belgium	2	30	30	30	30	30
72	Europe/Belgium/Antwerpen	1	32	32	32	32	32
73	Europe/Belgium/Beersel	1	30	30	30	30	30
74	Europe/Belgium/Brabant Wallon	1	30	30	30	30	30
75	Europe/Belgium/Brussels	2	32	32	32	32	32
76	Europe/Belgium/Brussels Capital Region	11	30	30	31	32	34
77	Europe/Belgium/Flemish Brabant	5	27	28	30	30	32
78	Europe/Belgium/Geel	1	30	30	30	30	30
79	Europe/Belgium/Ghent	3	29	30	30	31	32
80	Europe/Belgium/Hainaut	29	26	30	30	31	33
81	Europe/Belgium/Jette	1	34	34	34	34	34
82	Europe/Belgium/Liege	1	31	31	31	31	31
83	Europe/Belgium/Limburg	1	32	32	32	32	32
84	Europe/Belgium/Meerhout	2	30	30	31	31	31
85	Europe/Belgium/Namur	1	34	34	34	34	34
86	Europe/Belgium/Putte	5	21	30	30	30	32
87	Europe/Belgium/Sint-agatha-berchem	1	25	25	25	25	25
88	Europe/Belgium/Sint-Agatha-Berchem	1	34	34	34	34	34
89	Europe/Belgium/Sint-Pieters-Leeuw	3	29	29	29	30	30
90	Europe/Belgium/Vlamertinge	1	28	28	28	28	28
91	Europe/Belgium/Wallonie/Hainaut/Enghien	1	32	32	32	32	32
92	Europe/Belgium/Wilrijk	1	30	30	30	30	30
93	Europe/Croatia/The City of Zagreb	1	32	32	32	32	32
94	Europe/Croatia/Zagreb County	1	32	32	32	32	32

95	Europe/Czech Republic/Liberec Region/Rynoltice	1	29	29	29	29	29
96	Europe/Czech Republic/South Moravian Region/Adamov	2	29	30	30	31	31
97	Europe/Czech Republic/South Moravian Region/Brno	1	27	27	27	27	27
98	Europe/Czech Republic/South Moravian Region/Slavkov u Brna	1	29	29	29	29	29
99	Europe/Denmark	1	30	30	30	30	30
100	Europe/Denmark/Hovedstaden	46	29	30	31	31	32
101	Europe/Denmark/Midtjylland	66	29	32	32	32	34
102	Europe/Denmark/Nordjylland	17	30	32	32	33	34
103	Europe/Denmark/Sjaelland	4	29	30	31	31	32
104	Europe/Denmark/Syddanmark	12	23	30	30	30	33
105	Europe/Finland	3	30	30	30	31	31
106	Europe/France/Auvergne-Rhone-Alpes/Clermont Ferrand	1	26	26	26	26	26
107	Europe/France/Auvergne-Rhone-Alpes/Haute-Savoie	2	29	29	29	29	29
108	Europe/France/Auvergne-Rhone-Alpes/Thonon les bains	1	30	30	30	30	30
109	Europe/France/Bretagne/Rennes	2	28	28	29	29	29
110	Europe/France/Centre-Val de Loire/Eure-et-Loir	1	30	30	30	30	30
111	Europe/France/Centre-Val de Loire/Luisant	1	29	29	29	29	29
112	Europe/France/Ile-de-France	6	30	31	31	31	32
113	Europe/France/Nouvelle-Aquitaine	1	27	27	27	27	27
114	Europe/France/Nouvelle-Aquitaine/Bordeaux	1	30	30	30	30	30

115	Europe/France/Pays de loire/La roche sur yon	1	29	29	29	29	29
116	Europe/France/Provence-Alpes-Cote d Azur	15	27	30	30	31	31
117	Europe/France/Provence-Alpes-Cote dâ€™Azur	3	29	30	30	30	30
118	Europe/France/Provence-Alpes-Cote dâ€™Azur/Alpes-Maritimes	1	31	31	31	31	31
119	Europe/Germany/Baden-Wurttemberg	6	30	31	32	32	35
120	Europe/Germany/Bavaria	18	29	29	30	32	33
121	Europe/Germany/Bavaria/Munich	23	24	30	30	30	33
122	Europe/Germany/Berlin	1	29	29	29	29	29
123	Europe/Germany/Berlin/Berlin	1	31	31	31	31	31
124	Europe/Germany/Brandenburg	3	29	29	29	30	31
125	Europe/Germany/Hamburg	7	28	29	30	31	32
126	Europe/Germany/Hesse	24	29	29	30	31	32
127	Europe/Germany/Lower Saxony	4	28	29	30	30	30
128	Europe/Germany/North Rhine-Westphalia	6	29	29	30	31	33
129	Europe/Germany/North Rhine-Westphalia/DÃ¼sseldorf Health department	3	27	27	27	27	27
130	Europe/Germany/Saxony	1	27	27	27	27	27
131	Europe/Germany/Schleswig-Holstein	3	30	30	30	31	31
132	Europe/Germany/Thuringia	1	32	32	32	32	32
133	Europe/Gibraltar	6	31	33	33	35	38
134	Europe/Greece	1	28	28	28	28	28
135	Europe/Ireland/Dublin	1	30	30	30	30	30
136	Europe/Ireland/Longford	1	30	30	30	30	30
137	Europe/Ireland/Meath	4	25	29	30	30	31
138	Europe/Italy/Calabria	1	30	30	30	30	30
139	Europe/Italy/Campania	3	29	32	35	46	56

140	Europe/Italy/Lombardia	2	27	28	29	30	31
141	Europe/Italy/Piemonte	2	36	37	38	38	39
142	Europe/Italy/Puglia	2	28	28	28	28	28
143	Europe/Italy/Trentino-Alto Adige	1	29	29	29	29	29
144	Europe/Liechtenstein	1	26	26	26	26	26
145	Europe/Netherlands/Gelderland	1	32	32	32	32	32
146	Europe/Netherlands/Noord-Holland	39	28	29	30	31	33
147	Europe/Netherlands/Noord-Holland/Amsterdam	21	29	29	31	32	34
148	Europe/Netherlands/Utrecht	1	29	29	29	29	29
149	Europe/Netherlands/Zeeland/Tholen	1	29	29	29	29	29
150	Europe/Norway/Oslo	19	29	29	29	29	30
151	Europe/Norway/Vestland	1	29	29	29	29	29
152	Europe/Norway/Viken	12	29	29	29	30	31
153	Europe/Portugal	23	21	29	31	31	32
154	Europe/Romania/Bucuresti	2	30	30	31	31	31
155	Europe/Russia/Moscow	2	29	29	29	29	29
156	Europe/Slovakia/Nitra	2	30	30	30	30	30
157	Europe/Slovakia/Presov	1	30	30	30	30	30
158	Europe/Spain/Albacete	3	30	31	31	31	31
159	Europe/Spain/Balearic Island	6	19	30	31	31	33
160	Europe/Spain/Basque Country/Donostia-San Sebastian	1	29	29	29	29	29
161	Europe/Spain/Castilla-La Mancha/Ciudad Real	5	28	31	31	32	32
162	Europe/Spain/Catalunya	10	30	31	31	32	34
163	Europe/Spain/Catalunya/Barcelona	1	30	30	30	30	30
164	Europe/Spain/Catalunya/Girona	1	32	32	32	32	32
165	Europe/Spain/Galicia/Vigo	2	32	32	33	33	33
166	Europe/Spain/Madrid	22	30	32	33	35	41
167	Europe/Sweden/Jonkopings lan	1	30	30	30	30	30

168	Europe/Sweden/Skane	1	31	31	31	31	31
169	Europe/Sweden/Stockholm	14	21	28	30	32	33
170	Europe/Switzerland/Aargau	2	30	30	31	31	31
171	Europe/Switzerland/Basel-Landschaft	6	30	30	31	31	31
172	Europe/Switzerland/Basel-Stadt	2	31	31	31	31	31
173	Europe/Switzerland/Bern	5	28	28	29	30	30
174	Europe/Switzerland/BL	3	31	31	31	31	31
175	Europe/Switzerland/BS	8	31	31	31	31	31
176	Europe/Switzerland/Geneva	1	29	29	29	29	29
177	Europe/Switzerland/Solothurn	1	31	31	31	31	31
178	Europe/Switzerland/Thurgau	1	24	24	24	24	24
179	Europe/Switzerland/Vaud	3	30	30	30	30	30
180	Europe/Switzerland/Zug	1	29	29	29	29	29
181	Europe/Switzerland/Zurich	16	28	30	31	32	38
182	Europe/Turkey	1	30	30	30	30	30
183	Europe/United Kingdom/England	1700	27	32	33	35	43
184	Europe/United Kingdom/Scotland	412	27	31	32	34	39
185	Europe/United Kingdom/Wales	9	31	32	33	34	36
186	North America/Canada/Alberta	10	24	29	31	33	33
187	North America/Canada/British Columbia	5	30	30	31	31	31
188	North America/Canada/Manitoba	5	30	30	30	30	31
189	North America/Canada/Ontario	29	23	30	31	32	41
190	North America/Canada/Quebec	1	30	30	30	30	30
191	North America/Canada/Saskatchewan	4	29	31	32	32	33
192	North America/Mexico/Mexico City	2	31	31	31	31	31
193	North America/Mexico/State of Mexico	1	32	32	32	32	32
194	North America/Puerto Rico	1	29	29	29	29	29
195	North America/USA/Alaska/Anchorage-Mat Su	1	24	24	24	24	24
196	North America/USA/Arizona/Maricopa County	5	31	31	31	35	42

197	North America/USA/Arizona/Pima County	1	33	33	33	33	33
198	North America/USA/California	4	30	30	30	30	31
199	North America/USA/California/Alameda County	4	26	29	30	30	30
200	North America/USA/California/Los Angeles County	2	30	31	31	32	32
201	North America/USA/California/San Francisco County	1	32	32	32	32	32
202	North America/USA/California/West Sacramento	1	31	31	31	31	31
203	North America/USA/Colorado	2	26	27	28	29	30
204	North America/USA/Connecticut	3	24	28	31	31	31
205	North America/USA/Connecticut/Fairfield	4	30	30	31	32	32
206	North America/USA/Connecticut/Farifield	1	30	30	30	30	30
207	North America/USA/Connecticut/Hartford County	1	30	30	30	30	30
208	North America/USA/Connecticut/New Haven	2	30	30	31	31	31
209	North America/USA/District of Columbia	1	23	23	23	23	23
210	North America/USA/Florida	2	30	30	30	30	30
211	North America/USA/Georgia	4	29	29	30	31	32
212	North America/USA/Hawaii	2	30	31	31	32	32
213	North America/USA/Hawaii/Honolulu County	12	30	30	30	31	32
214	North America/USA/Idaho	1	30	30	30	30	30
215	North America/USA/Illinois/Cook County/Chicago	1	30	30	30	30	30
216	North America/USA/Iowa	1	31	31	31	31	31
217	North America/USA/Louisiana	2	29	30	31	31	32
218	North America/USA/Louisiana/Caddo Parish	1	31	31	31	31	31

219	North America/USA/Louisiana/Orleans	1	32	32	32	32	32
220	North America/USA/Louisiana/St Tammany	2	27	27	27	27	27
221	North America/USA/Maryland	10	27	30	30	30	31
222	North America/USA/Massachusetts	16	29	30	30	30	32
223	North America/USA/Massachusetts/Middlesex County	3	30	30	30	30	30
224	North America/USA/Michigan	1	31	31	31	31	31
225	North America/USA/Minnesota	2	30	30	30	30	30
226	North America/USA/Mississippi	1	29	29	29	29	29
227	North America/USA/Missouri	1	30	30	30	30	30
228	North America/USA/Nebraska	6	30	31	31	31	31
229	North America/USA/New Jersey	2	29	30	30	31	31
230	North America/USA/New York	5	29	30	30	30	31
231	North America/USA/New York/New York City	10	28	29	30	31	31
232	North America/USA/New York/Oneida	2	23	24	25	26	27
233	North America/USA/New York/Suffolk	2	25	26	27	28	29
234	North America/USA/New York/Westchester	1	31	31	31	31	31
235	North America/USA/Ohio	4	30	30	31	32	32
236	North America/USA/Oregon/Multnomah County	1	32	32	32	32	32
237	North America/USA/Oregon/Washington County	2	30	30	31	31	31
238	North America/USA/Pennsylvania	2	28	29	30	30	31
239	North America/USA/Rhode Island	1	30	30	30	30	30
240	North America/USA/Tennessee	1	23	23	23	23	23
241	North America/USA/Texas	4	31	32	32	32	32
242	North America/USA/Texas/Ellis	1	32	32	32	32	32

243	North America/USA/Texas/Houston	6	29	32	34	34	35
244	North America/USA/Texas/Houston County	25	32	32	33	34	44
245	North America/USA/Texas/San Antonio County	2	34	35	35	36	36
246	North America/USA/Texas/Tarrant County	1	26	26	26	26	26
247	North America/USA/Utah	4	28	30	31	31	31
248	North America/USA/Virginia	1	30	30	30	30	30
249	North America/USA/Washington	7	29	30	30	32	32
250	North America/USA/Washington/King County	1	30	30	30	30	30
251	North America/USA/Washington/Whatcom County	1	32	32	32	32	32
252	North America/USA/Wisconsin	1	32	32	32	32	32
253	North America/USA/Wisconsin/Milwaukee County	4	24	29	30	31	33
254	Oceania/Australia/Australian Capital Territory	10	29	30	30	30	31
255	Oceania/Australia/New South Wales/Sydney	79	19	28	30	30	32
256	Oceania/Australia/Northern Territory	1	29	29	29	29	29
257	Oceania/Australia/Queensland	2	33	35	37	38	40
258	South America/Argentina/San Luis	1	30	30	30	30	30
259	South America/Brazil/Distrito Federal/Brasilia	1	31	31	31	31	31
260	South America/Brazil/Federal District/Brasilia	1	30	30	30	30	30
261	South America/Brazil/Rio Grande do Sul/Santa Cruz do Sul	1	30	30	30	30	30

262	South America/Brazil/SÃ£o Paulo	1	30	30	30	30	30
263	South America/Brazil/Sao Paulo	2	29	29	29	29	29
264	South America/Brazil/Sao Paulo/Guarulhos	1	30	30	30	30	30
265	South America/Brazil/Sao Paulo/Limeira	1	30	30	30	30	30

Table S2

**Continent**	**O1**	**O2**	**O3**	**O4**	**O5**	**O6**	**total**
Africa	453 (43.73)	94 (9.07)	142 (13.71)	40 (3.86)	301 (29.05)	6 (0.58)	1036
Asia	84 (62.22)	17 (12.59)	20 (14.81)	3 (2.22)	11 (8.15)		135
Europe	279 (10.06)	173 (6.24)	2210 (79.7)	8 (0.29)	101 (3.64)	2 (0.07)	2773
North America	61 (24.3)	60 (23.9)	95 (37.85)	1 (0.4)	29 (11.55)	5 (1.99)	251
Oceania	5 (5.43)	5 (5.43)	10 (10.87)	1 (1.09)	70 (76.09)	1 (1.09)	92
South America	6 (66.67)	1 (11.11)	1 (11.11)		1 (11.11)		9

Table S3

Emerging Polymutant	R346		A701		I1081		N1192	
Amino acids	K	R	A	V	I	V	N	S
Total N	347	3949	3595	696	4132	164	4292	4
Africa/Botswana	1 (0.29)	78 (1.98)	79 (2.2)		79 (1.91)		79 (1.84)	
Africa/Ghana	10 (2.88)	23 (0.58)	33 (0.92)		33 (0.8)		33 (0.77)	
Africa/Malawi		3 (0.08)	3 (0.08)		3 (0.07)		3 (0.07)	
Africa/Nigeria	3 (0.86)	8 (0.2)	11 (0.31)		11 (0.27)		11 (0.26)	
Africa/Reunion	1 (0.29)	1 (0.03)	2 (0.06)		2 (0.05)		2 (0.05)	
Africa/Senegal		7 (0.18)	7 (0.19)		4 (0.1)	3 (1.83)	7 (0.16)	
Africa/Sierra Leone	1 (0.29)		1 (0.03)		1 (0.02)		1 (0.02)	
Africa/South Africa	31 (8.93)	869 (22.01)	848 (23.59)	52 (7.47)	900 (21.78)		900 (20.97)	
Asia/Bangladesh		2 (0.05)	2 (0.06)		2 (0.05)		2 (0.05)	
Asia/Hong Kong	6 (1.73)	12 (0.3)	16 (0.45)	2 (0.29)	18 (0.44)		16 (0.37)	2 (50)
Asia/India	1 (0.29)	6 (0.15)	7 (0.19)		7 (0.17)		7 (0.16)	
Asia/Israel	4 (1.15)	63 (1.6)	65 (1.81)	2 (0.29)	67 (1.62)		66 (1.54)	1 (25)
Asia/Japan	2 (0.58)	5 (0.13)	7 (0.19)		7 (0.17)		7 (0.16)	
Asia/Jordan		2 (0.05)	2 (0.06)		2 (0.05)		2 (0.05)	
Asia/Malaysia		1 (0.03)	1 (0.03)		1 (0.02)		1 (0.02)	
Asia/Maldives		1 (0.03)	1 (0.03)		1 (0.02)		1 (0.02)	
Asia/Nepal		2 (0.05)	2 (0.06)		2 (0.05)		2 (0.05)	
Asia/Pakistan		1 (0.03)	1 (0.03)		1 (0.02)		1 (0.02)	
Asia/Singapore	1 (0.29)	12 (0.3)	13 (0.36)		11 (0.27)	2 (1.22)	13 (0.3)	
Asia/South Korea		9 (0.23)	9 (0.25)		9 (0.22)		9 (0.21)	
Asia/Sri Lanka		2 (0.05)	2 (0.06)		2 (0.05)		2 (0.05)	
Asia/Thailand		3 (0.08)	3 (0.08)		3 (0.07)		3 (0.07)	
Europe/Austria	2 (0.58)	15 (0.38)	15 (0.42)	2 (0.29)	17 (0.41)		17 (0.4)	
Europe/Belgium	4 (1.15)	71 (1.8)	75 (2.09)		75 (1.82)		75 (1.75)	
Europe/Croatia		2 (0.05)	2 (0.06)		2 (0.05)		2 (0.05)	

Europe/Czech Republic		5 (0.13)	3 (0.08)	2 (0.29)	5 (0.12)		5 (0.12)	
Europe/Denmark	2 (0.58)	144 (3.65)	103 (2.87)	43 (6.18)	146 (3.53)		146 (3.4)	
Europe/Finland		3 (0.08)	3 (0.08)		3 (0.07)		3 (0.07)	
Europe/France	4 (1.15)	32 (0.81)	34 (0.95)	2 (0.29)	36 (0.87)		36 (0.84)	
Europe/Germany	3 (0.86)	98 (2.48)	93 (2.59)	8 (1.15)	101 (2.44)		101 (2.35)	
Europe/Gibraltar		6 (0.15)	6 (0.17)		6 (0.15)		6 (0.14)	
Europe/Greece		1 (0.03)	1 (0.03)		1 (0.02)		1 (0.02)	
Europe/Ireland		6 (0.15)	5 (0.14)	1 (0.14)	6 (0.15)		6 (0.14)	
Europe/Italy		11 (0.28)	10 (0.28)	1 (0.14)	11 (0.27)		11 (0.26)	
Europe/Liechtenstein		1 (0.03)	1 (0.03)		1 (0.02)		1 (0.02)	
Europe/Netherlands	6 (1.73)	57 (1.44)	59 (1.64)	4 (0.57)	63 (1.52)		63 (1.47)	
Europe/Norway	2 (0.58)	30 (0.76)	32 (0.89)		32 (0.77)		32 (0.75)	
Europe/Portugal		23 (0.58)	23 (0.64)		23 (0.56)		23 (0.54)	
Europe/Romania		2 (0.05)	2 (0.06)		2 (0.05)		2 (0.05)	
Europe/Russia		2 (0.05)	2 (0.06)		2 (0.05)		2 (0.05)	
Europe/Slovakia		3 (0.08)	3 (0.08)		3 (0.07)		3 (0.07)	
Europe/Spain	5 (1.44)	46 (1.16)	50 (1.39)	1 (0.14)	51 (1.23)		51 (1.19)	
Europe/Sweden		16 (0.41)	16 (0.45)		16 (0.39)		16 (0.37)	
Europe/Switzerland	4 (1.15)	45 (1.14)	49 (1.36)		49 (1.19)		49 (1.14)	
Europe/Turkey		1 (0.03)	1 (0.03)		1 (0.02)		1 (0.02)	
Europe/United Kingdom*	153 (44.09)	1968 (49.84)	1558 (43.34)	558 (80.17)	1962 (47.48)	159 (96.95)	2120 (49.39)	1 (25)
North America/Canada	17 (4.9)	37 (0.94)	54 (1.5)		54 (1.31)		54 (1.26)	
North America/Mexico		3 (0.08)	3 (0.08)		3 (0.07)		3 (0.07)	
North America/Puerto Rico		1 (0.03)	1 (0.03)		1 (0.02)		1 (0.02)	
North America/USA	36 (10.37)	157 (3.98)	176 (4.9)	17 (2.44)	193 (4.67)		193 (4.5)	
Oceania/Australia	48 (13.83)	44 (1.11)	92 (2.56)		92 (2.23)		92 (2.14)	
South America/Argentina		1 (0.03)		1 (0.14)	1 (0.02)		1 (0.02)	
South America/Brazil		8 (0.2)	8 (0.22)		8 (0.19)		8 (0.19)	

* there are five missing nucleotide observations

Table S4

Country	nsp3-V1069		nsp4-V94		RNA poly.-Y460		RNA poly.-L694		ORF3a-L106		N-D343	
	I	V	A	V	C	Y	I	L	F	L	D	G
Freq	1085	3206	1005	3290	3	4293	1685	2611	444	3852	3854	442
Africa/Botswana	8 (0.74)	71 (2.21)		79 (2.4)		79 (1.84)		79 (3.03)		79 (2.05)	79 (2.05)	
Africa/Ghana	3 (0.28)	30 (0.94)		33 (1)		33 (0.77)		33 (1.26)	5 (1.13)	28 (0.73)	28 (0.73)	5 (1.13)
Africa/Malawi		3 (0.09)		3 (0.09)		3 (0.07)		3 (0.11)		3 (0.08)	3 (0.08)	
Africa/Nigeria		11 (0.34)		11 (0.33)		11 (0.26)		11 (0.42)	1 (0.23)	10 (0.26)	10 (0.26)	1 (0.23)
Africa/Reunion		2 (0.06)		2 (0.06)		2 (0.05)		2 (0.08)		2 (0.05)	2 (0.05)	
Africa/Senegal		7 (0.22)		7 (0.21)		7 (0.16)		7 (0.27)	3 (0.68)	4 (0.1)	4 (0.1)	3 (0.68)
Africa/Sierra Leone		1 (0.03)		1 (0.03)		1 (0.02)		1 (0.04)		1 (0.03)	1 (0.03)	
Africa/South Africa	190 (17.51)	710 (22.15)		900 (27.36)		900 (20.96)		900 (34.47)	2 (0.45)	898 (23.31)	898 (23.3)	2 (0.45)
Asia/Bangladesh		2 (0.06)		2 (0.06)		2 (0.05)		2 (0.08)		2 (0.05)	2 (0.05)	
Asia/Hong Kong	2 (0.18)	16 (0.5)		18 (0.55)		18 (0.42)		18 (0.69)		18 (0.47)	18 (0.47)	
Asia/India	1 (0.09)	6 (0.19)		7 (0.21)		7 (0.16)		7 (0.27)		7 (0.18)	7 (0.18)	
Asia/Israel	8 (0.74)	59 (1.84)		67 (2.04)		67 (1.56)		67 (2.57)	6 (1.35)	61 (1.58)	63 (1.63)	4 (0.9)
Asia/Japan		7 (0.22)		7 (0.21)		7 (0.16)		7 (0.27)		7 (0.18)	7 (0.18)	
Asia/Jordan		2 (0.06)		2 (0.06)		2 (0.05)		2 (0.08)		2 (0.05)	2 (0.05)	
Asia/Malaysia		1 (0.03)		1 (0.03)		1 (0.02)		1 (0.04)		1 (0.03)	1 (0.03)	
Asia/Maldives		1 (0.03)		1 (0.03)		1 (0.02)		1 (0.04)		1 (0.03)	1 (0.03)	
Asia/Nepal		2 (0.06)		2 (0.06)		2 (0.05)		2 (0.08)		2 (0.05)	2 (0.05)	
Asia/Pakistan		1 (0.03)		1 (0.03)		1 (0.02)		1 (0.04)		1 (0.03)	1 (0.03)	
Asia/Singapore		13 (0.41)		13 (0.4)		13 (0.3)		13 (0.5)	2 (0.45)	11 (0.29)	11 (0.29)	2 (0.45)
Asia/South Korea		9 (0.28)		9 (0.27)		9 (0.21)		9 (0.34)		9 (0.23)	9 (0.23)	
Asia/Sri Lanka		2 (0.06)		2 (0.06)		2 (0.05)		2 (0.08)	2 (0.45)			2 (0.45)
Asia/Thailand	1 (0.09)	2 (0.06)		3 (0.09)		3 (0.07)		3 (0.11)	1 (0.23)	2 (0.05)	2 (0.05)	1 (0.23)
Europe/Austria	9 (0.83)	8 (0.25)		17 (0.52)		17 (0.4)		17 (0.65)		17 (0.44)	17 (0.44)	

Europe/Belgium	2 (0.18)	73 (2.28)		75 (2.28)	75 (1.75)		75 (2.87)		75 (1.95)	75 (1.95)	
Europe/Croatia	2 (0.18)			2 (0.06)	2 (0.05)		2 (0.08)		2 (0.05)	2 (0.05)	
Europe/Czech Republic	2 (0.18)	3 (0.09)		5 (0.15)	5 (0.12)		5 (0.19)		5 (0.13)	5 (0.13)	
Europe/Denmark	78 (7.19)	68 (2.12)		146 (4.44)	146 (3.4)		146 (5.59)	3 (0.68)	143 (3.71)	143 (3.71)	3 (0.68)
Europe/Finland	1 (0.09)	2 (0.06)		3 (0.09)	3 (0.07)		3 (0.11)		3 (0.08)	3 (0.08)	
Europe/France	19 (1.75)	17 (0.53)		36 (1.09)	36 (0.84)	3 (0.18)	33 (1.26)		36 (0.93)	36 (0.93)	
Europe/Germany	27 (2.49)	74 (2.31)		101 (3.07)	101 (2.35)		101 (3.87)	7 (1.58)	94 (2.44)	94 (2.44)	7 (1.58)
Europe/Gibraltar		6 (0.19)		6 (0.18)	6 (0.14)		6 (0.23)	5 (1.13)	1 (0.03)	1 (0.03)	5 (1.13)
Europe/Greece		1 (0.03)		1 (0.03)	1 (0.02)		1 (0.04)		1 (0.03)	1 (0.03)	
Europe/Ireland	1 (0.09)	5 (0.16)		6 (0.18)	6 (0.14)		6 (0.23)	1 (0.23)	5 (0.13)	5 (0.13)	1 (0.23)
Europe/Italy	1 (0.09)	10 (0.31)		11 (0.33)	11 (0.26)		11 (0.42)		11 (0.29)	11 (0.29)	
Europe/Liechtenstein		1 (0.03)		1 (0.03)	1 (0.02)		1 (0.04)		1 (0.03)	1 (0.03)	
Europe/Netherlands	7 (0.65)	56 (1.75)		63 (1.91)	63 (1.47)		63 (2.41)	4 (0.9)	59 (1.53)	59 (1.53)	4 (0.9)
Europe/Norway		32 (1)		32 (0.97)	32 (0.75)		32 (1.23)		32 (0.83)	32 (0.83)	
Europe/Portugal		23 (0.72)		23 (0.7)	23 (0.54)		23 (0.88)		23 (0.6)	23 (0.6)	
Europe/Romania	1 (0.09)	1 (0.03)		2 (0.06)	2 (0.05)		2 (0.08)		2 (0.05)	2 (0.05)	
Europe/Russia		2 (0.06)		2 (0.06)	2 (0.05)		2 (0.08)		2 (0.05)	2 (0.05)	
Europe/Slovakia		3 (0.09)		3 (0.09)	3 (0.07)		3 (0.11)		3 (0.08)	3 (0.08)	
Europe/Spain	34 (3.13)	17 (0.53)		51 (1.55)	51 (1.19)		51 (1.95)		51 (1.32)	51 (1.32)	
Europe/Sweden	1 (0.09)	15 (0.47)		16 (0.49)	16 (0.37)		16 (0.61)	7 (1.58)	9 (0.23)	9 (0.23)	7 (1.58)
Europe/Switzerland	4 (0.37)	45 (1.4)		49 (1.49)	49 (1.14)		49 (1.88)		49 (1.27)	49 (1.27)	
Europe/Turkey		1 (0.03)		1 (0.03)	1 (0.02)		1 (0.04)		1 (0.03)	1 (0.03)	
Europe/United Kingdom*	623 (57.42)	1493 (46.57)	1005 (100)	1115 (33.89)	2121 (49.41)	1681 (99.76)	440 (16.85)	335 (75.45)	1786 (46.37)	1786 (46.34)	335 (75.79)

North America/Canada	2 (0.18)	52 (1.62)	54 (1.64)		54 (1.26)		54 (2.07)	9 (2.03)	45 (1.17)	44 (1.14)	10 (2.26)
North America/Mexico		3 (0.09)	3 (0.09)		3 (0.07)		3 (0.11)		3 (0.08)	3 (0.08)	
North America/Puerto Rico		1 (0.03)	1 (0.03)		1 (0.02)		1 (0.04)		1 (0.03)	1 (0.03)	
North America/USA	32 (2.95)	161 (5.02)	193 (5.87)		193 (4.5)	1 (0.06)	192 (7.35)	50 (11.26)	143 (3.71)	144 (3.74)	49 (11.09)
Oceania/Australia	23 (2.12)	69 (2.15)	92 (2.8)	3 (100)	89 (2.07)		92 (3.52)	1 (0.23)	91 (2.36)	91 (2.36)	1 (0.23)
South America/Argentina	1 (0.09)		1 (0.03)		1 (0.02)		1 (0.04)		1 (0.03)	1 (0.03)	
South America/Brazil	2 (0.18)	6 (0.19)	8 (0.24)		8 (0.19)		8 (0.31)		8 (0.21)	8 (0.21)	
South America/Brazil		8 (0.2)									

* missing nucleotides

Table S5

Core haplotypes of spike and other genes	Frequency	S-A67	S-G339	S-R346	S-S477	S-T478	S-E484	S-Q493	S-G496	S-Q498	S-N501	S-T547	S-D614	S-H655	S-N679	S-P681	S-N764	S-D796	S-N856	S-Q954	S-N969	S-L981	S-I1081	nsp3.K38	nsp3-A1892	nsp4-T492	nsp5-P132	nsp5-V247	nsp5-T280	nsp5-S284	nsp6-I189	RdRp-P323	nsp14-I42	E-T9	M-D3	M-Q19	M-A63	N-P13	N-R203	N-G204
	REF	V	D	R	N	K	A	R	S	R	Y	K	G	Y	K	H	K	Y	K	H	K	F	I	R	T	I	H	V	T	S	V	I	V	I	G	E	T	L	K	R
VDRNKARSRYKGYKHKYKHKFI-RTIHVTSVIVIGETLKR	3530	.	.	.	.	.	.	.	.	.	.	.	.	.	.	.	.	.	.	.	.	.	.	.	.	.	.	.	.	.	.	.	.	.	.	.	.	.	.	.
VDKNKARSRYKGYKHKYKHKFI-RTIHVTSVIVIGETLKR	330	.	.	K	.	.	.	.	.	.	.	.	.	.	.	.	.	.	.	.	.	.	.	.	.	.	.	.	.	.	.	.	.	.	.	.	.	.	.	.
VDRNKARSRYKGYKHKYKHKFV-RTIHVTSVIVIGETLKR	163	.	.	.	.	.	.	.	.	.	.	.	.	.	.	.	.	.	.	.	.	.	V	.	.	.	.	.	.	.	.	.	.	.	.	.	.	.	.	.
ZZZZZZZZZZZZZZZZZZZZZZ-RTIHVTSVIVIGETLKR	66	Z	Z	Z	Z	Z	Z	Z	Z	Z	Z	Z	Z	Z	Z	Z	Z	Z	Z	Z	Z	Z	Z	.	.	.	.	.	.	.	.	.	.	.	.	.	.	.	.	.
VDRNKARSRYKGYKHKYKHKFI-RTIHVTSVIVIDETLKR	35	.	.	.	.	.	.	.	.	.	.	.	.	.	.	.	.	.	.	.	.	.	.	.	.	.	.	.	.	.	.	.	.	.	D	.	.	.	.	.
VDRNKARSRYKGYKHKYKHKFI-ZZZZZZZZZZZZZZZZZ	30	.	.	.	.	.	.	.	.	.	.	.	.	.	.	.	.	.	.	.	.	.	.	Z	Z	Z	Z	Z	Z	Z	Z	Z	Z	Z	Z	Z	Z	Z	Z	Z
ZZZZZZZZZZZZZZZZZZZZZZ-ZZZZZZZZZZZZZZZZZ	16	Z	Z	Z	Z	Z	Z	Z	Z	Z	Z	Z	Z	Z	Z	Z	Z	Z	Z	Z	Z	Z	Z	Z	Z	Z	Z	Z	Z	Z	Z	Z	Z	Z	Z	Z	Z	Z	Z	Z
VDRNKARSRYKGYKHKYKHKFI-RTIHVTSVIVIGETPKR	15	.	.	.	.	.	.	.	.	.	.	.	.	.	.	.	.	.	.	.	.	.	.	.	.	.	.	.	.	.	.	.	.	.	.	.	.	P	.	.
VGRNKARSRYKGYKHKYKHKFI-RTIHVTSVIVIGETLKR	12	.	G	.	.	.	.	.	.	.	.	.	.	.	.	.	.	.	.	.	.	.	.	.	.	.	.	.	.	.	.	.	.	.	.	.	.	.	.	.
ZZZZZZZZZZZZZZZZZZZZZZ-RTIHVTSVIVIDETLKR	11	Z	Z	Z	Z	Z	Z	Z	Z	Z	Z	Z	Z	Z	Z	Z	Z	Z	Z	Z	Z	Z	Z	.	.	.	.	.	.	.	.	.	.	.	D	.	.	.	.	.
VDRNKARSRYKGYKHNYKHKFI-RTIHVTSVIVIGETLKR	8	.	.	.	.	.	.	.	.	.	.	.	.	.	.	.	N	.	.	.	.	.	.	.	.	.	.	.	.	.	.	.	.	.	.	.	.	.	.	.
ADRNKARGRYTGYKHKYNHKLI-KAIHVTSITVIDETLKR	7	A	.	.	.	.	.	.	G	.	.	T	.	.	.	.	.	.	N	.	.	L	.	K	A	.	.	.	.	.	I	T	.	.	D	.	.	.	.	.
VDRNKARGRYTGYKHKYNHKLI-KAIHVTSITVIDETLKR	6	.	.	.	.	.	.	.	G	.	.	T	.	.	.	.	.	.	N	.	.	L	.	K	A	.	.	.	.	.	I	T	.	.	D	.	.	.	.	.
VDRSKEQGQNKGYKHKYKHKFI-RTIHVTSVIVIGETLKR	6	.	.	.	S	.	E	Q	G	Q	N	.	.	.	.	.	.	.	.	.	.	.	.	.	.	.	.	.	.	.	.	.	.	.	.	.	.	.	.	.
VDRNKARGQYKGYKHKYKHKFI-RTIHVTSVIVIGETLKR	5	.	.	.	.	.	.	.	G	Q	.	.	.	.	.	.	.	.	.	.	.	.	.	.	.	.	.	.	.	.	.	.	.	.	.	.	.	.	.	.
VDRNKARSRYKGYKHKYKHKFI-RTIYVTSVIVIGETLKR	5	.	.	.	.	.	.	.	.	.	.	.	.	.	.	.	.	.	.	.	.	.	.	.	.	.	Y	.	.	.	.	.	.	.	.	.	.	.	.	.
VDRNKARSRYKGYKHNYKHKFI-RTIHVTSVIVIGEALKR	5	.	.	.	.	.	.	.	.	.	.	.	.	.	.	.	N	.	.	.	.	.	.	.	.	.	.	.	.	.	.	.	.	.	.	.	A	.	.	.
VDRNKVRSRYKGYKHKYKHKFI-RTIHVTSVIVIGETLKR	5	.	.	.	.	.	V	.	.	.	.	.	.	.	.	.	.	.	.	.	.	.	.	.	.	.	.	.	.	.	.	.	.	.	.	.	.	.	.	.
Rare variable haplotypes of core	41	.	.	.	.	.	.	.	.	.	.	.	.	.	.	.	.	.	.	.	.	.	.	.	.	.	P	.	.	.	.	.	.	.	D	.	.	.	.	.

Table S6

Continent/Country/Region/Sub-region	2021-10-17	2021-11-23	2021-11-24	2021-11-25	2021-11-26	2021-11-27	2021-11-28	2021-11-29	2021-11-30	2021-12-01	2021-12-02	2021-12-03	2021-12-04	2021-12-05	2021-12-06	2021-12-07	2021-12-09	total
Africa / Ghana / Accra			1	4														5
Africa / Nigeria / Abuja	1																	1
Africa / South Africa / Western Cape Province / Cape Town Metro								2										2
Asia / Israel													3	1				4
Asia / Sri Lanka / Marawila			1									1						2
Asia / Thailand / Bangkok / Nongkhaem			1															1
Europe / Denmark / Midtjylland												1						1
Europe / Denmark / Syddanmark							2											2
Europe / Germany / Bavaria								5										5
Europe / Germany / Bavaria / Munich															1			1
Europe / Germany / North Rhine-Westphalia								1										1
Europe / Gibraltar															1	4		5
Europe / Ireland / Meath					1													1
Europe / Netherlands / Noord-Holland																	1	1
Europe / Netherlands / Noord-Holland / Amsterdam															3			3
Europe / Sweden / Stockholm								1		1				1	4			7
Europe / United Kingdom / England			2	1	4				1	1	2	2	2	3	3			21
Europe / United Kingdom / Scotland							1		1	1	1		2	2	5	14		27
North America / Canada / Alberta								1				3						4
North America / Canada / Ontario		1																1

North America / USA / California / San Francisco County							1											1
North America / USA / Connecticut / Fairfield														1				1
North America / USA / Georgia									1									1
North America / USA / Hawaii									1									1
North America / USA / Hawaii / Honolulu County												2						2
North America / USA / Louisiana															1			1
North America / USA / Louisiana / Caddo Parish															1			1
North America / USA / Louisiana / Orleans												1						1
North America / USA / Louisiana / St Tammany										2								2
North America / USA / Massachusetts										1								1
North America / USA / Ohio																1		1
North America / USA / Texas									1	1		1						3
North America / USA / Texas / Houston															4	1		5
North America / USA / Texas / Houston County									2	4	3	4	2		2	4		21
North America / USA / Texas / San Antonio County						1											1	2
North America / USA / Washington															2			2
North America / USA / Washington / Whatcom County										1								1
North America / USA / Wisconsin / Milwaukee County											1							1
Oceania / Australia / Queensland															1			1
**total**	1	1	5	5	5	1	4	10	7	12	7	15	9	8	28	24	2	144

Table S7

	England	Scotland	Wales
2021-12-01	4		
2021-12-02	5		
2021-12-03	4		
2021-12-04	13		
2021-12-05	7		
2021-12-06	17	1	
2021-12-07	8		1
